# Essential Oil-Bearing Plants From Balkan Peninsula: Promising Sources for New Drug Candidates for the Prevention and Treatment of Diabetes Mellitus and Dyslipidemia

**DOI:** 10.3389/fphar.2020.00989

**Published:** 2020-06-30

**Authors:** Simona Codruta Heghes, Lorena Filip, Oliviu Vostinaru, Cristina Mogosan, Doina Miere, Cristina Adela Iuga, Mirela Moldovan

**Affiliations:** ^1^Department of Drug Analysis, Iuliu Hatieganu University of Medicine and Pharmacy, Cluj-Napoca, Romania; ^2^Department of Bromatology, Hygiene, Nutrition, Iuliu Hatieganu University of Medicine and Pharmacy, Cluj-Napoca, Romania; ^3^Department of Pharmacology, Physiology and Physiopathology, Iuliu Hatieganu University of Medicine and Pharmacy, Cluj-Napoca, Romania; ^4^Department of Dermopharmacy and Cosmetics, Iuliu Hatieganu University of Medicine and Pharmacy, Cluj-Napoca, Romania

**Keywords:** essential oils, Balkans, terpenoids, antidiabetic, antihyperlipidemic, metabolic diseases

## Abstract

Metabolic diseases like diabetes mellitus or dyslipidemia have a complex etiology characterized by the interference of genetic predisposition and environmental factors like diet or lifestyle. Over time they can cause significant vascular complications, leading to dysfunction or failure of key organs (brain, heart), with possible fatal consequences or a severe reduction of life quality. Although current authorized drugs may successfully control blood glucose or cholesterol level, their use is often associated with severe side effects, therefore the development of new drug candidates is necessary for a better management of metabolic diseases. Among potential new drug sources, aromatic plants rich in essential oils like *Melissa officinalis* L., *Mentha x piperita* L., *Cuminum cyminum* L. or *Pistacia lentiscus* L. var. *chia* are very promising due to their diverse chemical composition and multiple mechanisms of action. This review describes a series of recent experimental studies investigating antidiabetic and hypolipemic effects of essential oils extracted from several aromatic plant species with an ethnopharmacological relevance in the Balkan peninsula. The pharmacological models used in the studies together with the putative mechanisms of action of the main constituents are also detailed. The presented data clearly sustain a potential administration of the studied essential oils for the prevention and treatment of metabolic diseases. Further research is needed in order to ascertain the therapeutic importance of these findings.

## Introduction

Metabolic diseases have a complex etiology dominated by genetic predisposition and environmental factors like diet or lifestyle (Barroso and McCarthy, 2019). The most important metabolic diseases with a significant impact on global health are diabetes mellitus (type 1 or insulin-dependent and type 2 or non-insulin-dependent) and dyslipidemias (hypercholesterolemia, hypertriglyceridemia, and mixed dyslipidemia). Diabetes mellitus affected at least 422 million people worldwide in 2014, and caused 1.5 million deaths in 2012, according to an official report of the World Health Organization (WHO) from 2016, which also stated that the global prevalence of the disease has nearly doubled from 1980, reaching an alarming level of 8.5% in the adult population (World Health Organization, 2016). Dyslipidemia is a major risk factor for ischemic heart disease and stroke, causing 2.6 million deaths and 29.7 million disability adjusted life years (DALYS) in 2008 (World Health Organization, 2008). In diabetes mellitus (DM), chronic hyperglycemia may result from an impaired insulin secretion and/or resistance of key peripheral tissues to the effects of insulin (Tan et al., 2019). In dyslipidemia, elevated concentrations of total cholesterol and low-density lipoproteins (LDL) can induce endothelial cell dysfunction and smooth muscle cell proliferation leading to the genesis of atherosclerotic plaque (Helkin et al., 2016). Over time, chronic hyperglycemia or hypercholesterolemia associated with the two diseases which can also coexist, can cause significant vascular complications, leading to dysfunction or failure of multiple organs (brain, heart, kidneys), with possible fatal consequences or a severe reduction of life quality (De Fronzo et al., 2015).

Despite recent advances in medical sciences and the development of multiple antidiabetic or antihyperlipidemic drug classes, the prevalence of diabetes mellitus and dyslipidemia and the associated risk of premature death have constantly increased in low- and medium-income countries (Magliano et al., 2019). The ever-growing treatment cost of metabolic diseases may have a huge economic impact in several parts of the world, limiting the access of patients to effective treatments. In this context, the discovery of new molecules of natural origin, capable of being translated into cost-effective antidiabetic and antihyperlipidemic drugs could be important for large categories of patients, having the potential advantages of being readily available and better tolerated (Yatoo et al., 2017).

In the Balkans and Eastern Mediterranean region, plants have been used in traditional medicine since Antiquity, for the treatment of various ailments of different organs and systems, including metabolic diseases. The influential works of Dioscorides or Galen highlighted the ethnopharmacological relevance of medicinal plants in the Greek and Roman civilizations, setting the foundations of modern medicine. In the last two decades, several active constituents of medicinal plants like flavonoids, saponins, alkaloids or coumarins have been extensively researched for the treatment of metabolic diseases, but essential oils were rarely studied for this purpose (Gushiken et al., 2016).

Essential oils are complex mixtures of aromatic terpenes (mainly monoterpenes and sesquiterpenes) formed as secondary metabolites in specialized secretory tissues of aromatic plants (Sharifi-Rad et al., 2017). Nowadays, essential oils as main constituents of aromatic plants, are widely used for their flavoring properties in food industry or as fragrances in cosmetology, but there is also a growing interest for the evaluation of their complex pharmacological effects. Multiple studies proved antioxidant, antimicrobial, anti-inflammatory, or antinociceptive effects of essential oils (Bakkali et al., 2008), but there are limited data concerning possible effects on glucose and lipid metabolism. Therefore, the aim of this review was to evaluate the antidiabetic and antihyperlipidemic effect of essential oils, presenting the experimental pharmacological models used for their study, with the subsequent mechanistic explanations.

## Essential Oil-Bearing Plants From the Balkans

### Ethnobotanical Data and Chemical Composition

Balkan peninsula is a vast territory extending from Central Europe in the north to the Eastern Mediterranean region in the south and from Black Sea in the east to the Adriatic Sea in the west. It includes the modern states of Albania, Bosnia-Herzegovina, Bulgaria, Croatia, Greece, Montenegro, North Macedonia, Romania (Dobrogea region), Serbia, Slovenia, and Turkey (European region). The complexity of Balkan geography favored a significant biodiversity in the plant kingdom (Griffiths et al., 2004), with a number of around 8,000 plant species and subspecies being catalogued, of which 2,600 to 2,700 are considered endemic (Stevanović et al., 2007). Not surprisingly, traditional medicine using plants for the treatment of various ailments, plays an important role in many Balkan countries, especially in remote, rural areas where access to healthcare system is difficult (Dzamic and Matejic, 2017). An ethnobotanical study from Bosnia-Herzegovina identified 228 plant species used in traditional medicine for the treatment of respiratory, gastrointestinal but also metabolic disorders, among which aromatic plants like *Foeniculum vulgare* Mill., or *Rosmarinus officinalis* L. are used in herbal infusions in rural areas from the central part of the country (Saric-Kundalic et al., 2010). In Greece, a field study found 109 plant species known for medicinal purposes, some of them being used for essential oil extraction, sometimes by traditional methods from aromatic plants cultivated in small areas like *Lavandula stoechas* L., used for skin infections and burns (Axiotis et al., 2018). Another study mentioned an endemic essential oil-bearing plant species, *Pistacia lentiscus* L. var. *chia*, uniquely present on Chios Island in the Eastern Aegean region of Greece, used in traditional medicine over the last 2,500 years in the treatment of gastrointestinal disorders which recently proved significant antihyperlipidemic and cardioprotective effects (Pachi et al., 2020). A study from Serbia identified 83 plant species including two aromatic plants present in the wild flora of the Kopaonik Mountain region (*Melissa officinalis* L. and *Thymus vulgaris* L.) which were used in the treatment of gastrointestinal, respiratory, or cardiac disorders but also in metabolic diseases (Jaric et al., 2007). In Turkey, a survey study found that of 64 plant species used in traditional medicine, 9 species were used for essential oil production which are usually sold in local markets as remedies for urinary, respiratory or cardiovascular diseases but also for diabetes like the essential oil from *Myrtus communis* L., endemic in the Mediterranean area (Gurdal and Kultur, 2013).

Our review of published data identified sixteen aromatic plant species, cultivated or wild in the Balkan region, belonging to nine families which presented antidiabetic and antihyperlipidemic properties demonstrated by experimental models. The plants were organized alphabetically by family and botanical name, the main chemical constituents being also presented (**Table 1**).

**Table 1:** Chemical composition of essential oils (EO) from Balkan region with antidiabetic and antihyperlipidemic activity.

No.	Plant species with essential oils (part used)	Main chemical constituents	C/W	References
1.	**Apiaceae** *Foeniculum vulgare* Mill. – fennel (seeds)	*trans-*anethole, 50.0–90.0%; limonene, 1.4–26.44%; γ-terpinene, 10.5%; α-pinene, 0.4–10.0%; 1,8-cineole, 1.0–6.0%	c	Miguel et al., 2010; Raal et al., 2012
2.	*Cuminum cyminum* L.—cumin (seeds)	cuminaldehyde, 19.25–27.02%; p-mentha-1,3-dien-7-al, 4.29–12.26%; γ-terpinene, 7.06–14.10%; p-cymene, 4.61–12.01%	c	Can Baser et al., 1992
	**Asteraceae**			
3.	*Tanacetum praeteritum* (Horw.) Heywood (flowers)	α-thujone, 0–79.4%; camphor, 0.7–37.6%; 1,8-cineole, 4.3–19.5%; bornyl acetate, 0–10.0%; terpinen-4-ol, 1.0–9.3%	w	Özek, 2018
	**Fabaceae**			
4.	*Trigonella foenum-graecum* L.—fenugreek (seeds)	neryl acetate, 17.32%; ß-pinene, 15.05%; β-caryophyllene, 14.63%; geranial, 4.81%; camphor, 16.32%	c	Hamden et al., 2011
	**Lamiaceae**			
5.	*Lavandula stoechas* L.—Spanish lavender (aerial part)	pulegone, 0–40.4%; α-pinene, 1.0–23.18%; camphor, 0–22.4%; menthol, 0–18.1%; menthone, 0–12.6%; lavandulyl acetate, 0–3.0%	c	Kirmizibekmez et al., 2009
6.	*Melissa officinalis* L.—lemon balm (leaves)	geranial, 0–65.42%; citronellal, 0.7–39.6%; neral, 3.28–31.5%; caryophyllene oxide, 0.2–10.26%; eugenol, 0.05–0.5%	w	Fahima et al., 2014
7.	*Mentha × piperita* L.—peppermint (aerial part)	menthol, 31.52%; menthone, 18.35%; carvone, 13.03%; isomenthol acetate, 7.63%; p-menthan-3-one, 6.21%	c	Abdellatief et al., 2017
8.	*Rosmarinus officinalis* L.—rosemary (aerial part)	α-pinene, 7.9–38.1%; verbenone, 15–37%; camphor, 1–22.35%; bornyl acetate, 0.9–12%	w	Satyal et al., 2017
9.	*Salvia sclarea* L.—clary sage (leaves)	germacrene D, 0.6–10.60%; geranyl acetate, 3.45–5.8%; neryl acetate, 1.8–3.0%; caryophyllene oxide, 0.50–2.2%	w	Souleles and Argyriadou, 1997
10.	*Thymus vulgaris* L.—common thyme (aerial part)	thymol, 30–48.2%; p-cymene, 2.2–42.8%; γ-terpinene, 0.3–30.90%; linalool, 1.3–12.4%; terpinen-4-ol, 0.3–9.5%; carvacrol, 0.5–5.5%	w	Borugă et al., 2014
	**Lauraceae**			
11.	*Laurus nobilis* L.—laurel, bay tree (leaves)	1,8 cineole, 24.2–68.82%; α-terpinenyl acetate, 4.8–18.65%; methyl eugenol, 0.2–16.7%; linalool, 0.7–16.0%; sabinene, 2.1–12.2%	w	Taban et al., 2018
	**Myrtaceae**			
12.	*Myrtus communis* L.—myrtle (leaves)	α-pinene, 8.1–56.7%; 1,8-cineole, 8–37%; myrtenyl acetate, 0.1–36%; limonene, 4.1–19%; linalool, 0.5–18.4%	w	Zomorodian et al., 2013
13.	**Pistaciaceae** *Pistacia lentiscus* L. var. *chia*—mastic tree (mastic gum)	α-pinene, 58.86–77.10%; myrcene, 0.23–12.27%; linalool, 0.45–3.71%; camphene, 0.75–1.04%	w	Papageorgiou et al., 1991
	**Ranunculaceae**			
14.	*Nigella sativa* L.—black cumin (seeds)	p-cymene, 18.46–52.64%; thymoquinone, 0.14–29.7%; carvacrol, 0.87–11.5%; α-terpineol, 5.11–9.72%	c	Ghanavi et al., 2018
	**Rutaceae**			
15.	*Citrus x aurantiifolia* (Christm.) Swingle—lime (leaves)	limonene, 57.84%; neral, 7.81%; linalool, 4.75%; isogeraniol, 3.48%; citronellal, 2.19%	c	Ibrahim et al., 2019
16.	*Citrus x limon* (L.) Osbeck—lemon (pericarps)	limonene, 53.07–80.0%; β-pinene, 9.53%; borneol, 5.57%; neral, 4.7%; sabinene, 4.18%; linalool, 3.70%	c	Oboh et al., 2017

C/W, cultivated/wild species.

**Figure 1:**
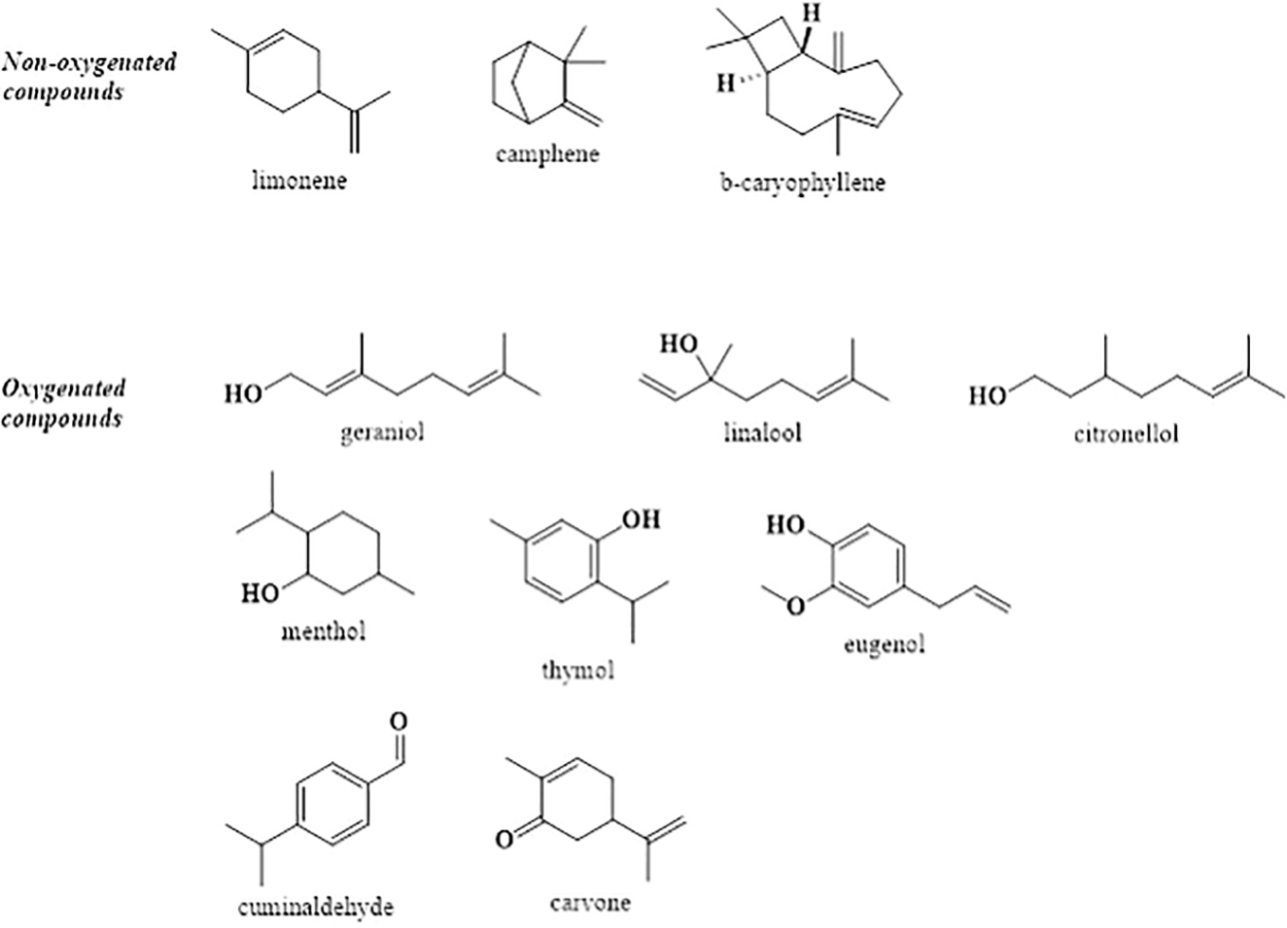
Chemical structures of main compounds with antidiabetic and antihyperlipidemic activity.

The concentration of main components from essential oils may be variable according to environmental conditions, plant chemotype or methods of harvesting. Also, other minor constituents from EOs could contribute to their biological effects. The most representative active compounds individually tested in several pharmacological models, are presented in **Figure 1**.

### Preclinical Studies Investigating Antidiabetic Effect of Essential Oils

This review showed that essential oils from fifteen aromatic plant species belonging to eight families presented antidiabetic properties demonstrated by specific *in vivo* or *in vitro* preclinical experimental models. The aromatic plant families with the highest proportion of species with antidiabetic essential oils (EO) were Lamiaceae (six species), Apiaceae (two species) and Rutaceae (two species). Other identified families were Asteraceae, Fabaceae, Lauraceae, Myrtaceae, and Ranunculaceae, each with only one plant species with antidiabetic essential oils (**Table 2**).

**Table 2:** Plant species containing essential oils (EO) with antidiabetic activity demonstrated in preclinical studies (*in vivo* and in *vitro*).

No.	Plant species with essential oils	Experimental model/Dose	Findings	References
1.	**Apiaceae** *Foeniculum vulgare* Mill.—fennel	Streptozotocin induced diabetes in rats/30 mg/kg EO	Reduction of hyperglycemia	Abou et al., 2011
2.	*Cuminum cyminum* L.—cumin	In vitro assessment of alpha-glucosidase In vitro assessment of insulin secretagogue action on isolated rat pancreatic islets	Inhibition of alpha-glucosidase with IC50 of 0.5 µg/ml 3.34-fold increase of insulin secretion	Lee, 2005 Patil et al., 2013
	**Asteraceae**			
3.	*Tanacetum praeteritum* (Horw.) Heywood	In vitro assessment of α-amylase	Inhibition of α-amylase with IC50 of 1.02 µg/ml	Özek, 2018
	**Fabaceae**			
4.	*Trigonella foenum-graecum* L.—fenugreek	Alloxan induced diabetes in rats/5% in food	Lowering of blood glucose, improvement of lipid profile, protective effect on pancreatic beta-cells	Hamden et al., 2011
	**Lamiaceae**			
5.	*Lavandula stoechas* L.—Spanish lavender	Alloxan induced diabetes in rats/50 mg/kg EO	Reduction of blood glucose, decrease of lipoperoxidation	Sebai et al., 2013
6.	*Melissa officinalis* L.—lemon balm	db/db mice model/0.015 mg/day EO	Reduction of blood glucose, improvement of glucose tolerance, increase of serum insulin, increase of glucokinase and GLUT-4 gene expression	Chung et al., 2010
7.	*Mentha × piperita* L.—peppermint	Streptozotocin induced diabetes in rats/40–80 mg/kg EO	Reduction of blood glucose, increase of serum insulin and C-peptide, upregulation of Bcl-2 expression	Abdellatief et al., 2017
8.	*Rosmarinus officinalis* L.—rosemary	Alloxan induced diabetes in rats/mg/kg EO	Reduction of blood glucose, hepatic and renal protective effects	Selmi et al., 2017
9.	*Salvia sclarea* L.—clary sage	Alloxan induced diabetes in mice/50–200 mg/kg EO	Reduction of blood glucose	Raafat and Habib, 2018
10.	*Thymus vulgaris* L.—common thyme	Alloxan induced diabetes in rats/0.4 ml/kg mg/kg EO	Reduction of blood glucose, improvement of lipid profile	Tuama, 2016
	**Lauraceae**			
11.	*Laurus nobilis* L. –laurel, bay tree	In vitro assessment of α-glucosidase	Inhibition of α-glucosidase with IC50 of 1.748 µg/ml	Sahin Basak and Candan, 2013
	**Myrtaceae**			
12.	*Myrtus communis* L.—myrtle	Alloxan induced diabetes in rabbits/50 mg/kg EO	Reduction of blood glucose, normalization of oral glucose tolerance test	Sepici et al., 2004
	**Ranunculaceae**			
13.	*Nigella sativa* L.—black cumin	Streptozotocin induced diabetes in rats/0.3% EO in diet	Reduction of blood glucose, increase of insulin serum concentration	Sultan et al., 2014
	**Rutaceae**			
14.	*Citrus × aurantiifolia* (Christm.) Swingle—lime	Alloxan induced diabetes in rats/50 ml/kg EO	Reduction of fasting blood and hepatic glucose, increase of glycogen in the liver	Ibrahim et al., 2019
15.	*Citrus x limon* (L.) Osbeck—lemon	In vitro assessment of α-amylase and α-glucosidase	Inhibition of α-amylase and α-glucosidase with IC50 of 8.16 and 7.56 µg/ml	Oboh et al., 2017

Previous reviews investigated the antidiabetic effects of natural compounds and medicinal plants. An extended study screened *in silico* a library of over 2,300 compounds derived from 30 common herb species, showing that liquorice, hops, fennel, and rosemary are a reach source of antidiabetic compounds like flavonoids and alkaloids (Perreira et al., 2019). Another study identified 111 medicinal plants who were reported to have a beneficial effect in diabetes mellitus, finding flavonoids, glycosides, and oligosaccharides as main active compounds (Eddouks et al., 2014). Our data confirmed some of the findings from these studies, demonstrating that α-amylase, α-glucosidase or glucose-6-phosphatase are effective molecular targets not only for the mentioned phytochemical classes but also for essential oils, equally capable of reducing blood glucose in a variety of experimental models.

The analysis of the aforementioned studies showed that the majority of experimental models used for the assessment of antidiabetic effect of essential oils were *in vivo* techniques (10 studies from 16). Among these, alloxan and streptozotocin-induced diabetes methods were preferred, being capable of reproducing the destruction of pancreatic beta-cells from diabetes with subsequent hyperglycemia and glucose intolerance. Alloxan enters the pancreatic beta cells where it suppresses ATP-mediated insulin release while streptozotocin alkylates the DNA of beta cells inducing their necrosis (Hamden et al., 2011). In both models, the administration of several essential oils reduced hyperglycemia and protected pancreatic beta cells against chemical aggression. Other methods employed genetically modified laboratory animals like *db/db* mice which can also reproduce some of the pathological features of diabetes mellitus. These mice have a mutation in their leptin receptor, with the subsequent development of insulin resistance and obesity which was partially corrected by the administration of lemon balm essential oil (Chung et al., 2010). Five experimental models used *in vitro* techniques for evaluating the effect of essential oils on important enzymes from glucose metabolism like α-amylase or α-glucosidase which are involved in the release of glucydic fractions in the digestive system (Özek, 2018). In these models, the administration of essential oils inhibited the two enzymes with IC50 ranging from 0.5 to 8.16 µg/ml. Although the experimental models used for the study of antidiabetic essential oils are quite diverse, having generated important data, further studies using additional techniques are necessary, for a more accurate evaluation.

### Preclinical Studies Investigating Antihyperlipidemic Effect of Essential Oils

This review showed that seven essential oils-bearing aromatic plant species belonging to five families presented antihyperlipidemic effects confirmed by specific *in vivo* or *in vitro* preclinical experimental models (**Table 3**).

**Table 3:** Plant species containing essential oils (EO) with antihyperlipidemic activity demonstrated in preclinical studies (*in vivo* and in *vitro*).

No.	Plant species with essential oils	Experimental model/Dose	Findings	References
1.	**Apiaceae** *Foeniculum vulgare* Mill.—fennel	Diet induced dyslipidemia in rats/30 mg/kg EO	Reduction of total cholesterol, LDL and TG, slight increase of HDL	Al-Okbi et al., 2018
2.	*Cuminum cyminum* L.—cumin	Diet induced dyslipidemia in rabbits/500 mg/kg EO	Reduction of total cholesterol, LDL and TG	Chouhan and Purohit, 2018
	**Lamiaceae**			
3.	*Melissa officinalis* L.—lemon balm	APOE2 transgenic mice In vitro assessment of cholesterol biosynthesis on HepG2 cells	Reduction of plasma TG Reduction of cellular cholesterol and TG	Jun et al., 2012
4.	*Rosmarinus officinalis* L.—rosemary	Diet induced dyslipidemia in rats/10 mg/kg EO	Reduction of total cholesterol, LDL and TG, slight increase of HDL	Al-Okbi et al., 2018
	**Pistaciaceae**			
5.	*Pistacia lentiscus* L. var. *chia*—mastic tree	Triton WR1339-induced dyslipidemia in rats In vitro assessment of cholesterol biosynthesis on HepG2 cells	Reduction of total cholesterol, LDL and TG Reduction of intracellular cholesterol biosynthesis	Vallianou et al., 2011
	**Ranunculaceae**			
6.	*Nigella sativa* L.—black cumin	Spontaneously hypertensive rats/800 mg/kg EO	Reduction of total cholesterol, LDL and TG	El-Dakhakhny et al., 2000
	**Rutaceae**			
7.	*Citrus x limon* (L.) Osbeck—lemon	Diet induced dyslipidemia in rabbits/250 mg/kg EO	Reduction of total cholesterol, LDL and TG; Reduction of aortic intima thickness	Lee et al., 2018

Six of the presented essential oils-bearing plants (fennel, cumin, lemon balm, rosemary, black cumin, and lemon) have also proved a priorly discussed antidiabetic activity, the co-existence of antidiabetic and antihyperlipidemic properties being a possible advantage in the development of new drug candidates. The presented data showed that predominantly, antihyperlipidemic effect of essential oil was evaluated using *in vivo* experiments in which dyslipidemia was induced either by a cholesterol-enriched diet or by the administration of Triton WR1339, an inhibitor of lipoprotein lipase which favors the accumulation of VLDL lipoproteins (Vallianou et al., 2011). In both models, the administration of the mentioned essential oils decreased the concentration of total cholesterol, LDL, and triglycerides. A study evaluated also the aortic intima thickness an important parameter in cardiovascular diseases, noting favorable effects after the administration of lemon essential oil for 8 weeks (Lee et al., 2018). Another experimental model used APOE2 transgenic mice, which develop hyperlipoproteinemia with elevated cholesterol and triglycerides. The administration of lemon balm essential oil produced in these animals a decrease of plasma triglycerides by inhibiting fatty acids synthesis following a down-regulation of Sterol Regulatory Element-Binding Protein-1c (SREBP-1c) (Jun et al., 2012). Also, two experimental models were performed *in vitro* using HepG2 cells, in which treatment with lemon balm and mastic tree essential oils reduced intracellular cholesterol biosynthesis. Due to the complexity of cholesterol metabolism, further studies are necessary to understand the molecular basis of antihyperlipidemic effect of essential oils.

### Clinical Studies With Essential Oils Used in Metabolic Diseases

The antidiabetic and antihyperlipidemic effects of selected essential oils were investigated in several clinical studies, summarized in **Table 4**. All selected studies used patients with diabetes, dyslipidemia, or metabolic syndrome, the essential oils being administered by oral route with a treatment duration ranging from 40 to 90 days.

**Table 4:** Clinical studies with antidiabetic and antihyperlipidemic essential oils.

Disease	Authors	Type of clinical study	Number of patients	Treatment	Results
Diabetes mellitus	Jafari et al., 2017	Randomized, control study	99 type 2 DM patients with suboptimal glycemic control	Cumin essential oil 50 and 100 mg/day for 8 weeks	Reduction of fasting blood glucose and glycosylated hemoglobin (HbA1C)
Diabetes mellitus	Bilal et al., 2009	Randomized control study	41 type 2 DM patients	Black cumin essential oil 700 mg/day for 40 days	Reduction of fasting blood glucose
Diabetes mellitus	Jafari et al., 2017	Randomized control study	64 prediabetic patients	Cumin essential oil 75 mg/day for 10 weeks	No significant change in glycemic indices; significant improvement of lipid profile
Diabetes mellitus, Dyslipidemia	Keihan et al., 2016	Randomized control study	95 diabetic and dyslipidemic patients	Cumin essential oil 25 mg/day for 90 days	Reduction of fasting blood glucose, HbA1C, triglycerides and oxidized LDL
Dyslipidemia	Hadi et al., 2018	Meta-analysis	6 RCTs with 376 patients	Cumin essential oil	Significant reduction of total cholesterol and LDL
Metabolic syndrome	Morovati et al., 2019	Randomized control study	56 patients with metabolic syndrome	Cumin essential oil 75 mg t.i.d. for 8 weeks	Reduction of fasting blood glucose and total cholesterol

The analysis of data resulted from the selected clinical studies showed that cumin (*Cuminum cyminum* L.*)* essential oil was predominantly used for the treatment of diabetes and dyslipidemia, only one study using another essential oil, extracted from the black cumin (*Nigella sativa* L.). According to the majority of these studies, the administered essential oils managed to reduce fasting blood glucose and glycosylated hemoglobin in diabetic patients, decreasing also total cholesterol and LDL concentration in dyslipidemia. Nevertheless, the presented clinical studies with antidiabetic and antihyperlipidemic essential oils have several limitations. They were represented mainly by randomized control studies (RCTs) using small numbers of patients with an insufficient statistical significance. Only one study was a meta-analysis with superior statistical power, although itself used a limited number of randomized controlled trials (Hadi et al., 2018). Therefore, additional clinical studies on larger populations are needed to ascertain the therapeutic value of essential oils with antidiabetic or antihyperlipidemic potential.

### Toxicological Evaluation of Essential Oils With Antidiabetic and Antihyperlipidemic Potential and Their Main Chemical Constituents

Essential oils are characterized by a high content in monoterpenes, compounds with low molecular weight and high lipophilicity which enable them to easily pass through biological barriers and potentially affect multiple organs. Despite their promising impact for health sciences, only a few studies investigated the toxicological aspects of essential oils with antidiabetic and antihyperlipidemic potential and their main chemical constituents. A study on female Wistar rats evaluated the repeated oral toxicity of essential oil from *Cuminum cyminum* L. and found no evidence of clinical signs of toxicity or pathological modifications at organ level, defining a non-observed adverse effect level (NOAEL) of 500 mg/kg/day (Taghizadeh et al., 2017). Another study evaluating the sub-chronic toxicity of DL-menthol in male B6C3F1 mice, also found no clinical signs or histopathological evidence of toxicity and established a NOAEL of 1956 mg/kg/day. In male Fischer 344 rats, the same study did not demonstrate significant signs of toxicity for DL-menthol, calculating a NOAEL of 937 mg/kg/day (OECD SIDS programs, 2003). The toxicity of eugenol was evaluated by an *in vitro* method using isolated rat hepatocytes, which demonstrated that an exposure to high doses of this compound present in several essential oils can cause significant hepatotoxicity, attenuated by acetylcysteine, showing some similarity with paracetamol intoxication (Nejad et al., 2017). For geraniol, a CYP-mediated metabolic pathway could generate geranial and 6,7-epoxygeraniol which proved significant sensitizing properties in the murine local lymph node assay (Hagvall et al., 2008).

In humans, several chemical constituents from essential oils like 1,8-cineole, and thujone have been shown to induce epileptiform convulsions, in children with a history of epileptic syndromes, according to a report (Burkhard et al., 1999). Additionally, the meta-analysis which investigated antidiabetic and antihyperlipidemic effects of cumin (*Cuminum cyminum* L.) essential oil in clinical trials, identified rare adverse effects like respiratory complications and dermatitis (Hadi et al., 2018). Due to insufficient and sometimes contradictory data, further research is needed to rigorously evaluate the safety profile of essential oils with antidiabetic and antihyperlipidemic potential and their main chemical constituents.

## Mechanisms of Action of Antidiabetic and Antihyperlipidemic Essential Oils With a Focus on Active Compounds

### Current Pharmacological Approach in Diabetes and Dyslipidemia

The main objective of antidiabetic medication is the reduction of hyperglycemia and the risk of vascular complications, which can be achieved by multiple pharmacological mechanisms, due to the complexity of glucose metabolism (Tan et al., 2019). Various types of insulins, with different pharmacokinetic properties are administered subcutaneously in type 1 diabetes, acting as agonists on specific receptors expressed by a variety of cells including hepatocytes, adipocytes, or muscular cells. Subsequently, key glucose transporters (GLUT) are activated at cellular level, inducing a significant reduction of blood glucose. For type 2 diabetes, multiple classes of antidiabetic drugs are currently on the market. Sulfonylureas and meglitinides act as insulin secretagogues, amplifying the exocytosis of insulin granules from pancreatic beta-cells. Other classes like biguanides increase the uptake and metabolism of glucose in peripheral tissues while inhibitors of alpha-glucosidase limit the amount of absorbed glucose in the digestive tract. Another approach was to target peroxisome proliferator-activated receptors gamma (PPAR-γ) with thiazolidinedione drugs, in order to obtain an increased expression of glucose transporters in key peripheral cells. The more recently developed incretin mimetic drugs can either act as agonists on incretin receptors or inhibit incretin metabolism by blocking dipeptidyl-peptidase IV enzyme, both with complex beneficial effects on glucose metabolism. Finally, the inhibitors of sodium-glucose cotransporter 2 (SGLT-2) act by augmenting the urinary excretion of glucose (De Fronzo et al., 2015; Tan et al., 2019).

In dyslipidemia, the main objective of the medication is to minimize the risk of major vascular events like stroke or heart attack by reducing the level of LDL, the most atherogenic lipoprotein fraction. The backbone of antihyperlipidemic drugs is represented by statins which inhibit 3-hydroxy-3-methylglutaryl-coenzyme A (HMG-CoA) reductase, reducing the formation of mevalonate, a precursor of cholesterol. Additionally, statins may increase the number of LDL receptors on hepatocytes which also contributes to their cholesterol lowering effect (Chang and Robidoux, 2017). Fibrates are used especially in hypertriglyceridemia and mixed dyslipidemia, acting as agonists on peroxisome proliferator-activated receptor-alpha (PPAR-alpha) which leads to the activation of lipoprotein lipase with the subsequent degradation of VLDL fraction rich in triglycerides. Bile acid sequestrants like cholestyramine act by removing bile acids from enterohepatic circuit with the slow development of a cholesterol lowering effect. Ezetimibe, a more recent drug, can reduce intestinal absorption of cholesterol by blocking its main transporter, the Nieman-Pick C1-Like1 (NPC1L1) protein, being frequently used in association with statins (Tiwari and Khokhar, 2014). A novel class is represented by monoclonal antibodies against proprotein convertase subtilisin/kexin type 9 (PCSK9), a protein involved in lysosomal degradation of LDL receptors in the hepatocytes. They are reserved for selected cases of familial heterozygous or homozygous hypercholesterolemia in patients insufficiently controlled with the maximal tolerable doses of statins (Toth et al., 2017).

Unfortunately, the beneficial effects of many existing antidiabetic or antihyperlipidemic drugs are accompanied by numerous adverse effects, sometimes severe, which can limit patient’s compliance and treatment efficacy. Hence, there is a growing need for new drug candidates with improved characteristics, among which naturally occurring molecules like essential oils could play an important part. Essential oils have a complex chemical composition which allows them to target multiple physiological structures and processes involved in glucose metabolism, several mechanisms of action being described. Although some aspects of the antidiabetic effect of essential oils and their constituents have been explained, further research is needed for a better understanding of their cellular and molecular actions.

### Antidiabetic Mechanisms of Essential Oils

#### Inhibition of Glucose Absorption From the Digestive Tract

Glucose is the main energy source in the human body, being obtained generally by degrading more complex molecules like starch, sucrose, or lactose. After the initial action of salivary and pancreatic α-amylase, the digestion is continued in the small intestine epithelium by α-glucosidase (along with maltase and lactase) with the release of absorbable glucose, fructose, or galactose. Therefore, inhibition of α-glucosidase and α-amylase can reduce the amount of absorbable glucydic fractions from the digestive tract, being capable of preventing postprandial hyperglycemic peaks. The mechanism is clinically validated for acarbose and miglitol, two α-glucosidase inhibitors which are used in the treatment of type 2 diabetic patients. A significant inhibitory effect on α-glucosidase was demonstrated *in vitro* for cuminaldehyde a main component in cumin essential oil. Cuminaldehide showed an IC50 value of 0.5 µg/ml, being only slightly inferior to acarbose, proving also a strong inhibitory effect on aldose reductase (IC50 of 0.085 µg/ml) which may suggest additional protective effects against toxic actions of sorbitol in diabetic neuropathy (Lee, 2005). Also, the inhibition of α-glucosidase was demonstrated by another *in vitro* study investigating the essential oil from *Laurus nobilis* L., which showed a 90% inhibition of the enzyme (IC50 of 1.748 µg/ml), the main chemical constituent responsible for the effect being 1,8-cineole (Sahin Basak and Candan, 2013). The research of Özek (2018) identified a significant inhibition of α-amylase for the essential oil from *Tanacetum praeteritum* subsp*. praeteritum* (Horw.) Heywood, in the I_2_/KI method with IC50 of 1.02 µg/ml, suggesting that **thujone** is the main component responsible for the effect.

#### Increase of Glycogen Synthesis in the Liver and Decrease of Gluconeogenesis

Glucose homeostasis is a multi-faceted process in which the liver has an important role, being capable of storing or releasing glucose. In diabetic patients, a decreased glycogen synthesis coupled with increased gluconeogenesis and glycogenolysis are detected, which lead to elevated blood glucose. Therefore, stimulation of glucokinase and more importantly glycogen synthase is capable of reducing hyperglycemia by stimulating the conversion of glucose into glycogen. The *in vivo* study of Ibrahim et al. (2019) proved that essential oil from *Citrus x aurantiifolia* (Christm.) Swingle presented a hypoglycemic effect in an alloxan-induced diabetes model. In this study, intraperitoneal administration of 100 mg/kg EO to diabetic rats for 14 days, significantly reduced fasting blood glucose levels, decreased hepatic glucose production, and increased hepatic glycogen, suggesting that reduction of gluconeogenesis and augmentation of glycogenesis are the main mechanism of action of the essential oil. On the other hand, a new *in vivo* study showed that oral administration of **geraniol** a natural monoterpene found in several essential oils, in doses between 100 and 400 mg/kg, to STZ-induced diabetic rats for 45 days, reduced plasma glucose, and glycosylated hemoglobin (HbA1C). The analysis of liver tissue from the treated rats proved that geraniol was capable of inhibiting hepatic glucose-6-phosphatase activity, thus reducing gluconeogenesis (Babukumar et al., 2017). Also, other three studies found that citronellol, d-limonene, and caryophyllene inhibited both glucose-6-phosphatase and fructose-1-6-phosphatase, reducing gluconeogenesis in STZ-induced diabetic rats, with a significant reduction of plasma glucose and HbA1C (Murali and Saravanan, 2012; Srinivasan and Muruganathan, 2016; Basha and Sankaranarayanan, 2014).

#### Increase of Insulin Sensitivity

Insulin intracellular signaling is a complex process finalized with the translocation of GLUT2 and GLUT4 glucose transporters from the cytoplasm of target cells to their membrane. Phosphatidylinositol 3-kinase (PI3K) and adenosine monophosphate-activated protein kinase (AMPK) contribute to this process. An *in vivo* study using genetically modified diabetic mice (db/db) demonstrated that oral administration of essential oil from *Melissa officinalis* L. in a dose of 0.015 mg/day for 6 weeks, significantly reduced blood glucose and improved glucose tolerance. The RT-PCR and Western blotting methods performed in this study proved that the essential oil was able to increase the expression of glucose transporter (GLUT4) genes probably by the activation of PI3K cascade, leading to an increased glucose uptake in key cells of treated animals (Chung et al., 2010). Also, an *in vitro* study on cultivated astrocytes originating from C57BL/6 mice pups, proved that **eugenol**, a main constituent in several essential oils, was capable of enhancing intracellular insulin signaling mechanism by increasing AKT phosphorylation, without changing PTP-1B expression (Sartorius et al., 2014).

#### Increase of Insulin Secretion

An increase of insulin secretion could be achieved by depolarizing beta cell membrane by modulating potassium channels or by protecting beta cells against aggression and apoptosis. An *in vivo* study proved that **menthol**, present in the chemical composition of several essential oils, administered orally to STZ-induced diabetic rats in doses of 25 to 100 mg/kg for 45 days, reduced blood glucose and glycosylated hemoglobin (HbA1C). The substance showed a mechanism of action similar with sulphonylurea drugs, being able to inhibit ATP-sensitive K channels on beta cells membrane, increasing the exocytosis of insulin. Additionally, menthol increased the survivability of beta cells by stimulating the expression of Bcl-2, an antiapoptotic factor (Muruganathan et al., 2017). The results were confirmed by Abdellatief et al. (2017) who demonstrated that essential oil from *Mentha x piperita* L. also increased the expression of Bcl-2 and hence protected pancreatic beta cells against apoptosis in a STZ-induced diabetes model in rats.

### Antihyperlipidemic Mechanisms of Essential Oils

#### Activation of Lipoprotein Lipase

Lipoprotein lipase (LPL) is capable of hydrolyzing triacylglycerols from VLDL fraction, with the apparition of monoacylglycerol used by specific tissues. The activation of the enzyme is produced by fibrates, a well-established class of drugs used in the treatment of hypertriglyceridemia. An *in vivo* study using Triton WR1339-induced dyslipidemia model in rats, proved that essential oil from the plant *Pistacia lentiscus* L. var. *chia*, endemic in the island of Chios in Greece, was capable of activating lipoprotein lipase in rats with a subsequent 34.5% reduction of triglycerides (Vallianou et al., 2011). The study showed also that **camphene** was the active substance responsible for this effect, no significant changes in plasma lipids of treated animals being observed for other terpenoids present in the essential oil, which were individually tested. Additionally, camphene reduced also total cholesterol and LDL, suggesting that this monoterpene could be a possible drug candidate in the development of new antihyperlipidemic molecules. Another *in vivo* study using high fed diet (HFD) mice found that administration of **thymol** (10–40 mg/kg for 5 weeks), an important active compound from thyme essential oil, caused a reduction of triglycerides and total cholesterol in treated animals, producing also an increase in HDL concentration. The study suggested that the augmentation of lipoprotein lipase (LPL) activity and a reduction in leptin concentration could be main mechanisms responsible for the antihyperlipidemic effect (Saravanan and Pari, 2015).

#### Modulation of TRPV1 Receptor

The transient receptor potential vanilloid 1 (TRPV1) was considered to have important roles on sensory neurons, however other research demonstrated its presence on hepatocytes with possible roles in reducing blood cholesterol level. A recently published study investigated the effects of **eugenol**, an active compound present in several essential oils, including lemon balm EO, on lipid profile in rats fed with cholesterol-enriched diet. The obtained data showed that **eugenol** administered orally to the rats, in doses of 10 to 100 mg/kg, reduced total cholesterol, LDL, and atherogenic index in treated animals. Immunohistochemical methods proved that menthol down-regulated TRPV1 receptor in the liver of treated animals, mechanism supported by collateral molecular docking studies. Additionally, a histopathological examination performed in the same study found that eugenol protected the liver, reducing hepatic steatosis and the level of alanine aminotransferase (ALT) and alkaline phosphatase (ALP) activity (Harb et al., 2019).

#### Reduction of Fatty Acid Synthesis by Modulation of SREBP-1c

Excessive production of VLDL in which fatty acid synthesis plays an important role is involved in dyslipidemia and metabolic syndrome. A key enzyme in the process is fatty acid synthase (FAS) which is regulated by complex nuclear mechanisms. An *in vivo* study investigating the effects of essential oil from *Melissa officinalis* L. in APOE2 transgenic mice proved a decrease of plasma triglycerides by inhibiting fatty acids synthesis. The obtained data showed that the treatment with essential oil inhibited the nuclear translocation of Sterol Regulatory Element-Binding Protein-1c (SREBP-1c) which in turn led to the suppression of key genes involved in lipid metabolism, primarily fatty acid synthase (FAS). The study did not investigate which of the essential oil active constituents could be responsible for this effect (Jun et al., 2012).

## Conclusion

This review identified sixteen essential oil-bearing plant species from Balkan region with antidiabetic and antihyperlipidemic effects demonstrated in preclinical and clinical studies. The main mechanisms of antidiabetic effect of individual chemical components identified in the selected species were represented by an inhibition of α-amylase and α-glucosidase by cuminaldehyde, 1,8-cineole, and thujone, a decrease of gluconeogenesis by geraniol and an increase of insulin sensitivity by eugenol or insulin secretion by menthol. Activation of lipoprotein lipase by camphene and thymol and modulation of TRPV1 receptor by eugenol were considered responsible for the antihyperlipidemic effect. The active terpenoid components identified in the chemical composition of the studied essential oils could become promising new drug candidates, but future studies are needed in order to evaluate their potential.

## Author Contributions

Conceptualization and methodology: SH, LF, and OV. Validation and formal analysis: CM, DM, CI, and MM.

## Conflict of Interest

The authors declare that the research was conducted in the absence of any commercial or financial relationships that could be construed as a potential conflict of interest.

The handling editor declared a shared affiliation, though no other collaboration, with the authors at the time of the review.
